# Second-Trimester Induction Abortion Using Two Pretreatment Mifepristone Regimens: A Randomized Controlled Trial

**DOI:** 10.7759/cureus.90455

**Published:** 2025-08-19

**Authors:** Vaishnavi Rajaraman, Sudha Sumathy, Drsobha Nair, Radhamany K, Greeshma C Ravindran

**Affiliations:** 1 Obstetrics and Gynecology, Amrita Institute of Medical Sciences, Amrita Vishwa Vidyapeetham Healthcare Campus, Kochi, IND; 2 Biostatistics, Amrita Institute of Medical Sciences, Amrita Vishwa Vidyapeetham Healthcare Campus, Kochi, IND

**Keywords:** iei, mifepristone, misoprostol, rct, second-trimester abortion

## Abstract

Background

Second-trimester abortion is a critical aspect of reproductive healthcare, and reducing the induction-to-expulsion interval (IEI) improves patient outcomes. Mifepristone followed by misoprostol is the standard regimen. This study evaluates whether sequential dosing of mifepristone shortens the IEI compared to a single dose.

Methods

A randomized controlled trial was conducted at Amrita Institute of Medical Sciences with 54 women between 13 and 20 weeks of gestation. Participants were randomized into two groups: the Mife-1 group received a single 200 mg dose of mifepristone on Day 1, while the Mife-2 group received 200 mg on Days 1 and 2. Misoprostol (600 mcg vaginally, followed by 400 mcg orally every four hours) was administered after 36-48 hours. The primary outcome was IEI; secondary outcomes included prostaglandin requirement and the need for surgical evacuation.

Results

The Mife-2 group had a significantly shorter IEI (7.93 ± 3.679 hours) compared with the Mife-1 group (10.41 ± 4.263 hours, p = 0.026). Prostaglandin requirement was lower in the Mife-2 group, though not statistically significant (p = 0.087). Fewer participants in the Mife-2 group required surgical evacuation.

Conclusions

Sequential mifepristone dosing significantly reduces IEI, suggesting improved efficiency in second-trimester abortion. Larger studies are recommended to confirm safety and effectiveness.

## Introduction

Second-trimester medical abortion is the lawful termination of pregnancy between 13 and 24 weeks of gestation. India was one of the first countries to legalize abortion, enacting the Medical Termination of Pregnancy (MTP) Act in 1971, which was amended in 2020 to address issues arising from the 15.6 million miscarriages occurring annually [1-3]. To achieve the global goal of reducing maternal mortality to fewer than 70 per 100,000 live births, preventing abortion-related deaths is crucial, as unsafe abortions account for 13% of maternal deaths, predominantly in the second trimester. Expanding access to safe abortion services is therefore essential [4,5].

Mid-trimester termination has become increasingly common with widespread prenatal screening, especially for pregnancies affected by major fetal abnormalities. However, medical abortion has limitations, including the need for hospitalization, manual evacuation, and the psychological impact of labor and delivery. In addition to the indications outlined in the MTP Act, most second-trimester terminations are performed due to fetal anomalies or severe intrauterine growth restriction with significant Doppler abnormalities detected in early mid-trimester pregnancies [6,7].

Methods for terminating mid-trimester pregnancies include surgical approaches, such as dilation and evacuation (D&E), or pharmacological induction. The widely accepted and WHO-recommended regimen is the combined use of mifepristone and misoprostol [8].

Mifepristone, a steroidal anti-progestin also known as RU 486, is primarily used as an abortifacient to terminate early pregnancy by blocking progesterone receptors essential for maintaining gestation. It is typically combined with prostaglandins to enhance uterine contractions and induce embryo expulsion. Mifepristone is rapidly absorbed, reaching peak serum concentrations within one to two hours of oral administration. It has a prolonged half-life of 25-30 hours, with bioavailability influenced by serum α1-acid glycoprotein (AAG), which binds the drug. Mifepristone undergoes extensive metabolism via demethylation and hydroxylation, primarily catalyzed by the cytochrome P450 enzyme CYP3A4, and its metabolites retain biological activity [9,10].

Our study aims to evaluate the induction-to-expulsion interval (IEI) after administering two sequential doses of mifepristone followed by misoprostol. We hypothesize that repeating the dose after its half-life will re-saturate AAG, increasing the availability of free mifepristone to diffuse into peripheral tissues and bind uterine progesterone receptors, thereby enhancing its abortifacient effect.

## Materials and methods

Study design

The present study is a randomized controlled trial conducted at Amrita Institute of Medical Sciences (AIMS), Kochi, India, following approval from the institutional ethics committee of AIMS. The trial was prospectively registered in the CTRI registry (registration number: CTRI/2022/09/045721) on September 21, 2022. We recruited 54 consecutive women admitted to the obstetrics and gynecology department for second-trimester termination of pregnancy, with gestational ages between 13 and 20 weeks, from October 2022 to June 2023, who met the inclusion criteria and provided informed consent.

**Figure 1:**
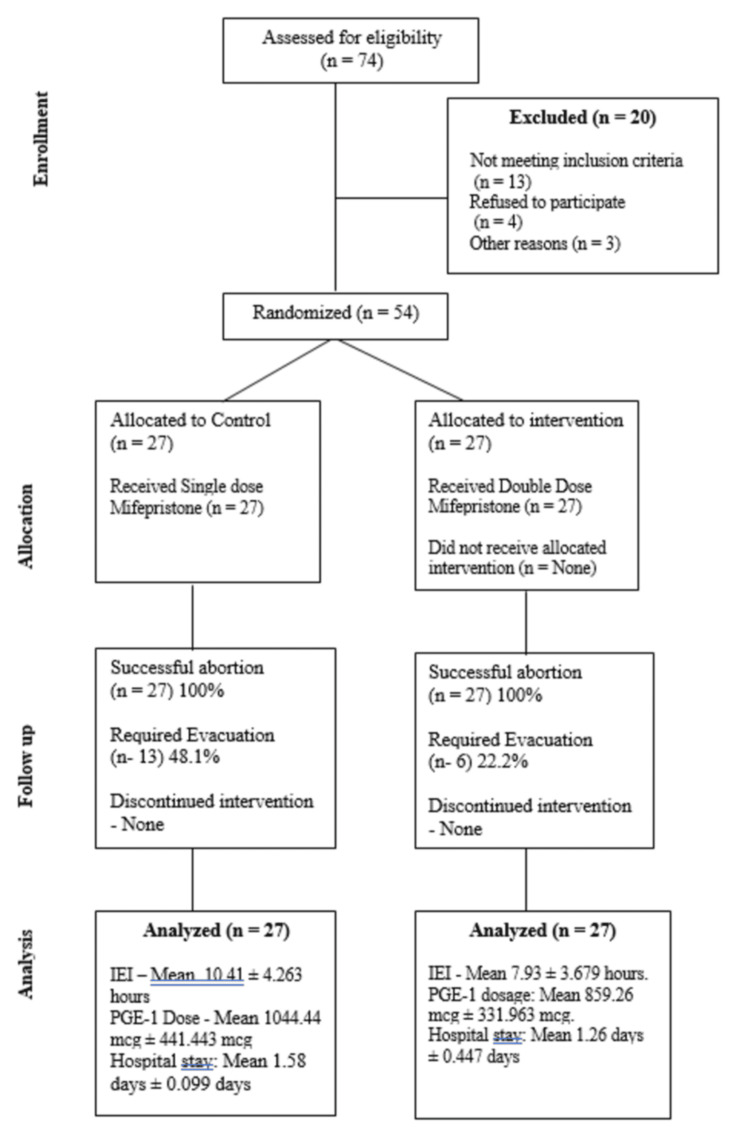
Completed CONSORT checklist for the randomized controlled trial CONSORT, CONsolidated Standards Of Reporting Trials

A completed CONSORT checklist for this randomized controlled trial is provided in Figure 1.

Technical information

Eligible women were between 13 and 20 weeks of gestation, with a live fetus in utero, a closed cervical os, no vaginal bleeding, and no contraindications to the study drugs or vaginal delivery. Written informed consent was obtained from all participants. A database was established to collect information on all women undergoing terminations during the study period.

Once the decision to terminate the pregnancy was made, the woman and her support person were referred to the medical termination unit, where pretermination counseling was conducted. The procedure, anticipated complications, and informed consent process were explained. Preprocedural evaluations included complete blood count, blood grouping and typing, and screening for viral markers.

Participants were randomly assigned to either the Mife-1 or Mife-2 group. The Mife-1 group received a single oral dose of mifepristone on Day 1, while the Mife-2 group received 200 mg of mifepristone orally on Day 1, followed by a second dose 24 hours later. After 36-48 hours of mifepristone administration, all patients were admitted to the labor care unit and given 600 mcg of vaginal misoprostol, followed by 400 mcg of oral prostaglandin E1 (PGE1) every four hours, for a maximum of four to five doses. Side effects, such as shivering, nausea, and vomiting, were recorded.

Successful termination of pregnancy was defined as complete expulsion of the products of conception within 24-48 hours of the first misoprostol dose. The expelled products (fetus and placenta) were examined for completeness. Parenteral tramadol was administered for analgesia. Oxytocin infusion (10 units in 500 ml of normal saline at 15-30 drops per minute) was started after fetal expulsion.

Surgical evacuation of the uterus was performed if there was evidence of retained placenta or incomplete abortion. The primary outcome was the IEI after initiation of PGE1, while secondary outcomes included the success rate of abortion, en sac expulsion, the need for evacuation, and PGE1 dosage requirements.

Anti-D immunoglobulin was administered to Rh-D-negative, non-sensitized women. Lactation suppression was achieved using cabergoline. Participants requiring evacuation of retained products received a single dose of parenteral cefuroxime. Contraceptive advice and birth-spacing counseling were provided. Women were discharged at the provider’s discretion after stabilization.

Statistical details

Based on pilot study results comparing the mean ± SD of IEI intervals in second-trimester abortions using two pretreatment mifepristone regimens (control group: 14.40 ± 4.775 hours; case group: 9.80 ± 6.496 hours; sample size: 5 per group), a minimum of 24 participants per group (total 48) was required to achieve 80% power with 95% confidence.

Statistical analysis was performed using IBM SPSS Statistics for Windows, Version 20.0 (Released 2011; IBM Corp., Armonk, NY, USA). Continuous variables are presented as mean ± SD, and categorical variables as frequency and percentage. The independent sample t-test was used to compare mean differences between groups, and the chi-square test was applied for associations between categorical variables. A p-value <0.05 was considered statistically significant.

## Results

The study included two groups: the Mife-1 group and the Mife-2 group, each comprising 27 women undergoing second-trimester abortion. Baseline parameters and outcomes were compared between the groups during the study period.

The mean age of participants was 29.96 ± 4.83 years in the Mife-1 group and 29.56 ± 5.91 years in the Mife-2 group (P = 0.615), indicating comparable age distribution. Parity distribution was similar across groups, with 10 women (37%) classified as nulliparous or primigravida. Additionally, 14 women (51.8%) in the Mife-1 group and 13 women (48.1%) in the Mife-2 group were multiparous without previous cesarean sections (CSs). Women with a history of CSs comprised three (11.1%) in the Mife-1 group and four (14.8%) in the Mife-2 group.

The mean gestational age was 18 weeks 5 days ± 2.1 in the Mife-1 group and 19 weeks 1 day ± 1.6 in the Mife-2 group (P = 0.362), demonstrating comparable gestational age distributions. Mean BMI was 24.015 ± 1.8581 kg/m² in the Mife-1 group and 23.944 ± 1.0405 kg/m² in the Mife-2 group (P = 0.972). Mean hemoglobin levels were 11.073 ± 0.6797 g/dl for the Mife-1 group and 11.281 ± 0.6000 g/dl for the Mife-2 group (P = 0.221).

The indication for abortion in both groups was fetal congenital malformation, with no significant difference between the groups (P = 1.000) (Table 1).

**Table 1:** Comparison of baseline characteristics between the Mife-1 and Mife-2 groups, including age, parity, gestational age, BMI, hemoglobin, and indications for abortion under the MTP Act Statistical significance was assessed using P-values, with P < 0.05 considered significant. Values are presented as N (%) for categorical variables and mean ± SD for continuous variables. CS, cesarean section; MTP, Medical Termination of Pregnancy

Baseline parameters	Group A	Group B	Chi-square value	P-value
Mean age in years ± SD	29.96 ± 4.829	29.56 ± 5.912	-	0.615
Range	18	22		
Parity				
Nullipara + primigravida	10 (37%)	10 (37%)	0.220	1.000
Multipara without previous CS	14 (51.8%)	13 (48.1%)		
Multipara with previous CS	3 (11.1%)	4 (14.8%)		
Mean gestation in weeks	18 weeks + 5 days ± 2.1	19 weeks + 1 day ± 1.6	-	0.362
13-18 weeks + 6 days	9 (33%)	6 (22.2%)		
19-20 weeks	18 (66.7%)	21 (77.8%)		
Mean BMI (kg/m²) ± SD	24.015 ± 1.8581	23.944 ± 1.0405	-	0.972
Range	9	4.4		
Mean hemoglobin (gm/dl) ± SD	11.073 ± 0.6797	11.281 ± 0.6000	-	0.221
Range	2.9	2.8		
Indication for abortion (Medical Termination of Pregnancy Act, Govt. of India, 1971)				
Fetal congenital malformation	27 (100%)	27 (100%)	-	1.000

Primary outcome

The success rate of abortion was 100% in both groups, indicating uniform efficacy across both treatment strategies (P = 1.000). A total of 64.81% of participants achieved complete abortion without secondary intervention, while 35.19% required surgical evacuation. Among those needing secondary intervention, 24.07% were from the Mife-1 group and 11.11% from the Mife-2 group.

Notably, the IEI was significantly shorter in the Mife-2 group, with a mean of 7.93 ± 3.679 hours compared to 10.41 ± 4.263 hours in the Mife-1 group (P = 0.026), indicating that sequential dosing in the Mife-2 group facilitated a faster abortion process.

Secondary outcomes

PGE1 Dosage

The mean PGE1 requirement was lower in the Mife-2 group (859.26 ± 331.96 µg) compared to the Mife-1 group (1044.44 ± 441.44 µg), though the difference was not statistically significant (P = 0.087). This trend suggests that sequential mifepristone dosing may reduce prostaglandin needs, likely due to enhanced progesterone receptor blockade from the second dose, which may improve uterine sensitivity to misoprostol and result in more efficient contractions.

Duration of Hospital Stay

The Mife-2 group had a significantly shorter hospital stay (1.26 ± 0.447 days) compared to the Mife-1 group (1.58 ± 0.099 days, P = 0.028). The shorter stay reflects the quicker abortion process facilitated by the reduced IEI, allowing for faster recovery and discharge.

Surgical Evacuation

A significantly higher proportion of women in the Mife-1 group required surgical evacuation (48.1%) compared to the Mife-2 group (22.2%) (P = 0.046), indicating greater efficacy of the sequential regimen. Evacuation was indicated when retained products of conception were detected on ultrasound after the maximum PGE1 doses. Diagnostic criteria for incomplete abortion included a heterogeneous echogenic mass within the endometrial cavity, endometrial thickness >15 mm, and possible Doppler vascularity. An enlarged uterus may also be seen. Complete abortion was defined by a thin endometrial stripe (<10 mm) with no residual tissue.

Analgesia Requirements

Analgesia was required more frequently in the Mife-1 group (51.9%) than in the Mife-2 group (37%), though the difference was not statistically significant (P = 0.411). This trend may be related to the longer IEI in the Mife-1 group, leading to extended discomfort and greater analgesic use. The shorter IEI in the Mife-2 group likely contributed to reduced pain duration and lower analgesia requirements (Table 2).

**Table 2:** Comparison of outcomes between the Mife-1 and Mife-2 groups, including success rates, IEI, PGE1 dosage, hospital stay, need for surgical evacuation, and analgesia requirement The IEI was significantly shorter in the Mife-2 group compared to the Mife-1 group (P = 0.026), and the duration of hospital stay was also significantly shorter in the Mife-2 group (P = 0.028). Statistical significance was assessed using P-values, with P < 0.05 considered significant. Values are presented as N (%) for categorical variables and mean ± SD for continuous variables. Bold values indicate statistical significance. IEI, induction-to-expulsion interval; PGE1, prostaglandin E1

Parameter	Group A	Group B	Chi-square value	P-value
Successful abortions	27 (100%)	27 (100%)	-	1.000
Induction to expulsion interval				
Mean ± SD (hours)	10.41 ± 4.263	7.93 ± 3.679	-	0.026
Range (min)	17	15		
PGE1 requirement				
Mean ± SD	1044.44 ± 441.443	859.26 ± 331.963	-	0.087
Range	1600	800		
Duration of hospital stay				
Mean ± SD	1.58 ± 0.099	1.26 ± 0.447	-	0.028
Range	1	1		
Need for evacuation	13 (48.1%)	6 (22.2%)	3.979	0.046
Need for analgesia	14 (51.9%)	10 (37%)	1.200	0.411

Overall, both groups achieved a 100% success rate for abortion; however, the Mife-2 group demonstrated a significantly shorter IEI, reduced hospital stay, and fewer surgical evacuations. These findings suggest that the sequential mifepristone regimen used in the Mife-2 group was more efficient and associated with better overall outcomes compared to the Mife-1 group (Table 3).

**Table 3:** Comparison of mean misoprostol doses between gestational age groups (13-18 weeks and 19-20 weeks) in the Mife-1 and Mife-2 groups Statistical significance was assessed using P-values, with P < 0.05 considered significant. Values are presented as mean ± SD.

Gestational age group (weeks)	N	Mean ± SD misoprostol dose (μg)	P-value	N	Mean ± SD misoprostol dose (μg)	P-value
		Group A			Group B	
13-18 weeks	9	886.7 ± 346.4	0.142	6	733.3 ± 326.6	0.301
19-20 weeks	18	1133.3 ± 465.3		21	895.2 ± 332.4	

## Discussion

Main findings

In the current study, the mean IEI was 10.41 ± 4.263 hours in the single-dose mifepristone group (Mife-1) compared to 7.93 ± 3.679 hours in the two-dose group (Mife-2), with a P-value of 0.026, indicating a statistically significant difference. The mean PGE1 requirement was 1044 ± 441 µg (three doses) in the Mife-1 group and 859 ± 331 µg (two doses) in the Mife-2 group (P = 0.087). Although the Mife-2 group required less PGE1, the difference was not statistically significant.

The mean duration of hospital stay was 1.56 ± 0.51 days in the Mife-1 group and 1.26 ± 0.45 days in the Mife-2 group (P = 0.027). The average pain score was 4.3 ± 1.04 out of 10 in the Mife-1 group and 3.67 ± 0.83 in the Mife-2 group, with analgesia provided as oral tramadol or paracetamol. Common side effects included shivering, nausea, and vomiting, all managed conservatively. Adverse drug effects occurred in eight patients (29.6%) in the Mife-1 group and four patients (14.8%) in the Mife-2 group.

Pain perception was subjective, and the literature on analgesic management during abortion care is limited, with heterogeneous data preventing robust comparison. Optimal analgesic strategies for first- and second-trimester terminations require further research [11].

Strengths

Despite the low incidence of mid-trimester abortions, our status as a tertiary care center with a fully equipped fetal medicine unit allowed us to gather a substantial number of cases.

Limitations

This single-center study was conducted in a tertiary teaching hospital. The relatively small sample size, due to the rarity of mid-trimester abortions, limits generalizability. Additionally, participants were only followed until the completion of the abortion procedure, without assessment of long-term outcomes such as complications or recovery time.

Interpretation

Our study contributes to the growing body of literature on the use of mifepristone and misoprostol for second-trimester abortions. Consistent with other studies, both regimens were highly effective in achieving successful termination of pregnancy, with a 100% success rate in both groups. This aligns with findings from Ashok and Templeton, who reported a 98% success rate for a combined regimen of mifepristone and misoprostol in second-trimester terminations, further supporting the reliability of this treatment approach [12].

The most notable finding in our study was the significant decrease in the IEI in the two-dose mifepristone regimen compared to the single-dose regimen. This is consistent with the study by Shantikumar et al., which also observed a reduction in IEI with a double-dose protocol. The shorter induction time in the Mife-2 group may be attributed to enhanced progesterone receptor saturation achieved by the second dose of mifepristone, which could potentiate the effects of misoprostol and lead to faster uterine contractions [13].

Compared to the literature, our findings support the reported efficacy of the mifepristone-misoprostol combination and further highlight that optimizing the mifepristone dosing regimen can improve the IEI.

However, according to Yang et al., using a higher dose of misoprostol can induce strong uterine contractions, which may increase the risk of serious complications such as uterine rupture or amniotic fluid embolism, particularly if the cervix is immature or the cervical canal is obstructed. Our study also indicates a lower requirement for PGE1 in the double-dose regimen, although this difference was not statistically significant. A smaller required dose could reduce dose-related complications [14].

While D&E of the uterus is a safe and effective technique, it can only be performed by experienced gynecologists. Even when conducted by experts, there remains a risk of severe complications, such as uterine perforation (0.4%) and cervical laceration (0.3-1.2%), as reported in the literature. Mentula et al. reported a 1.7% incidence of uterine perforation; in contrast, no cases occurred in our study [15].

Additionally, Webster et al. and Ashok et al. reported incidences of blood transfusion of 2.8% and 0.7%, respectively. In our study, two participants experienced bleeding slightly above normal limits, which was controlled with pharmacological agents, and no transfusions were required. Ashok et al. also reported one case (0.1%) of laparotomy for a myometrial tear. In contrast, our study encountered no severe complications such as hysterotomy, blood transfusion, or the need for cervical or vaginal laceration repairs [12,16].

Considering these findings, it is important to reflect on the broader implications for clinical practice and policymaking [17]. Overall, this study underscores the potential benefits of a double-dose mifepristone regimen, which may be more efficient while maintaining a similar or potentially lower risk of complications, consistent with previous studies.

## Conclusions

Two sequential doses of mifepristone followed by misoprostol significantly improve second-trimester abortion outcomes by reducing the IEI, hospital stay, and the need for surgical intervention. This regimen shows promise for broader clinical use, although further studies are needed to evaluate long-term safety and effectiveness across diverse patient populations.
